# Supplementary material for: Genetic Evaluation and Population Structure of Jiangsu Native Pigs in China Revealed by SINE Insertion Polymorphisms
Source: Animals (Basel). 2022 May 25;12(11):1345. doi: 10.3390/ani12111345 (PMC9179424; doi:10.3390/ani12111345)
Supplement: Supplementary file 1 [file animals-12-01345-s001.zip › animals-1681307-supplementary.pdf]

Table S1 Origin and sample size of Jiangsu pig populations, commercial breeds and Italic native breed

Breed or population	Origin	Samples
EHL		24
MMS	Suzhou, Jiangsu	32
FJ		29
SMS	Zhenjiang, Jiangsu	30
SWT	Haimen, Jiangsu	32
M	Jingtan, Jiangsu	32
H	Donghai, Jiangsu	23
SJ	Taizhou, Jiangsu	32
SS	Jingjiang, Jiangsu	32
LW	Xuzhou, Jiangsu	32
DRC		32
SB	Sicilian, Italia	32

Note: EHL, Erhualian; FJ, Fengjing; MMS, Middle Meishan; M, Mi; SWT, Shawutou; SJ, Sujiang; SS, Sushan; SMS, Small meishan; H, Huai; SB, Sicilian black pig; LW, Large White; DRC, Duroc.

Table S2. Primers of the 18 SINE-RIPs markers

Number	Primer	Primer sequence	Location	Tm/℃	Predicted PCR product size (bp)
1	REF-815	F:TCAGCCTGTTTCTCTTGGTCA<br>R:TCTGGGTTTCCTCTGTGCAT	chr1:78702902-78702903	58	412/696
2	REF-12270	F:CTTTTGTCCTTCACTGTTCATCA<br>R:TCTGCGTTGTGTCCACTCTA	chr2:132104789-132104790	58	450/713
3	REF-13182	F:AACGCTGGATCCTTAACCCA<br>R:TGGTGGCAGGTAGAAGTATGT	chr3:53759982-53759983	58	432/727
4	REF-14427	F:ACAACACAAGCCCCAAATGA<br>R:TGACTTTTCTGTGTTGGTCTTGT	chr4:79318339-79318340	58	400/650
5	REF-16131	F:GCCACCTGCCACAATATACA<br>R:AGGAAAAGCAATCTGACTGGA	chr5:74984667-74984668	58	432/732
6	REF-16684	F:TCTTAACCCACTGTGCCACA<br>R:TGGCTTCCTTAAATATCTGTGGG	chr6:92016839-92016840	58	432/722

7	REF-18327	F:CAGATGAGGTTGAGCTGTGC<br>R:GTTTGTCCCTCTCTCCCACT	chr7:59715296-59715297	58	411/617
8	REF-19717	F:ACACAGGTTTGAGAGCAGAC<br>R:GTTGACCTTCTGCCTCATGG	chr8:85085953-85085954	58	434/706
9	REF-21609	F:GAATGCCACTTTCCCCACAA<br>R:TCAGTGAGTAGGTGGCAGAG	chr9:122993573-122993574	58	411/693
10	REF-2929	F:CCAACTGCATGCTCTTCCAG<br>R:TCCTGATTATCTTGGAAATGGCT	chr10:59802734-59802735	58	445/739
11	REF-3719	F:TTCTCTTCCCTTCCTGACCG<br>R:CATGTTGGTACCCCTCCCAT	chr11:60869980-60869981	58	402/711
12	REF-4531	F:AGCTTTCCCTCTTTCTCCGT<br>R:CCGTCCATATTGCCAAGTCG	chr12:47951373-47951374	58	440/737
13	REF-5597	F:AACCAAAGCAGTGTTCAGGG<br>R:AGGGTTTGGGATGATGATGGT	chr13:106226341-106226342	58	417/710
14	REF-7445	F:TCTAGCTTTTCTTACCATTGGCT<br>R:ATCCAGTGGCTAGGCTAGAC	chr14:125956814-125956815	58	410/673
15	REF-8430	F:ACCACACAAGGCACATTTTG<br>R:GGCCTCTTTATTCACCCTCCT	chr15:97783631-97783632	58	417/631
16	REF-9435	F:GCCTGTGTCAGTACTTCATTCA<br>R:TGGGGTTAACAGATACACACTAC	chr16:55631725-55631726	58	443/669
17	REF-10096	F:CTCGCCCCTTACTTCAGACA<br>R:AACCATCACCACTGAACCCC	chr17:30485010-30485011	58	410/672
18	REF-11062	F:AGTCTCCCACTCACATTGCC<br>R:CCTCTGAGCTGCTCTTCCTT	chr18:50578198-50578199	58	400/690
